# Photonic topological transitions for surface waves with resonant plasmonic metasurfaces: interplay between meta-atom and lattice stretching

**DOI:** 10.1038/s41598-026-50214-w

**Published:** 2026-05-07

**Authors:** Artem Hrinchenko, Veronika Batianova, Sergey Polevoy, Oleh Yermakov

**Affiliations:** 1 V. N. Karazin Kharkiv National University, Kharkiv, Ukraine; 2 Abbe School of Photonics, Friedrich Schiller University, Jena, Germany; 3 Department of Radiospectroscopy, O. Ya. Usikov Institute for Radiophysics and Electronics of the NASU, Kharkiv, Ukraine; 4 Department of Fiber Photonics, Leibniz Institute of Photonic Technology, Jena, Germany

**Keywords:** Photonics, Plasmonics, Photonic topological transition, Metasurfaces, Hyperbolic metamaterials, Plasmon canalization, Materials science, Nanoscience and technology, Optics and photonics, Physics

## Abstract

The optical resonances of plasmonic metasurfaces can have a significant impact on the ability to control the light properties. In particular, resonant plasmonic metasurfaces exhibit photonic topological transitions, leading to sharp transformations in the topology of the isofrequency contours in reciprocal lattice space. For instance, elliptical contours can transform into hyperbolic ones, and vice versa, significantly altering the wavefront and properties of the propagating light. This transition is accompanied by the flat isofrequency contours corresponding to the canalization regime, which means the divergenceless and high-directional wave propagation. In this work, we investigate the formation and engineering of the photonic topological transitions and the related effects in the plasmonic metasurfaces based on gold nanopatches via the continuous transformation from isotropic to anisotropic cases. We study the impact of the induced anisotropy on the spectral positions and amplitudes of the surface conductivity resonances describing the metasurfaces leading to the emergence of hyperbolicity, near-field hot-spots and plasmon canalization. Particular attention is paid to a comparative analysis of the two anisotropy-inducing strategies, stretching the meta-atoms and stretching the lattice, in terms of functionality and fabrication of plasmonic resonant metasurfaces. Our findings emphasize the importance of using anisotropic resonant nanostructures for a plethora of photonic applications, especially in the area of the in-plane light management.

**Supplementary Information:**

The online version contains supplementary material available at 10.1038/s41598-026-50214-w.

## Introduction

In strongly anisotropic optical media and systems, the topology of the isofrequency contour in wave-vector space can change from closed to open, and vice versa, denoting the *photonic topological transition*^1^. The common dispersion relation in the non-magnetic uniaxial medium described by the permittivity tensor $\varepsilon = \text {diag} \left( \varepsilon _{\Vert }, \varepsilon _{\Vert }, \varepsilon _{\perp } \right)$ can be written as^2^:

1 $$\begin{aligned} \frac{k_x^2 + k_y^2}{\varepsilon _{\perp }} + \frac{k_z^2}{\varepsilon _{\Vert }} = k_0^2, \end{aligned}$$

where $k_i$ are the components of the wave vector $\textbf{k}$, $k_0 = 2 \pi / \lambda$ is the wavevector of plane wave in vacuum, $\lambda$ is the operational wavelength. At the fixed angular frequency, the surface in three-dimensional $\textbf{k}$-space is ellipsoid for $\varepsilon _{\Vert } \varepsilon _{\perp } > 0$ and hyperboloid for $\varepsilon _{\Vert } \varepsilon _{\perp } < 0$. In two-dimensional space, the isofrequency contours are elliptical and hyperbolic, respectively. The photonic topological transition, i.e. the transition from the closed elliptical to open hyperbolic contour (or vice versa), is accompanied by the change of the sign for one of the principal component of permittivity tensor. Besides, the epsilon-near-zero regime^3,4^ taking place in the vicinity of photonic topological transition is also characterized by the *canalization* regime, also known as self-collimation, channeling and tunneling^5,6^. The canalization means the self-collimating divergenceless propagation along the specific direction and is characterized by the flat isofrequency contour. So, the photonic topological transitions can be defined as the elliptical-flat-hyperbola transformations of isofrequency contours and are usually found in metamaterials^1^.

The resulting reconfigurations of the isofrequency contours dramatically impact on the light properties including the group and phase velocities, propagation direction, fields structure, photonic density of states, and strongly modify light–matter interactions, especially in the near field. The metamaterials characterized by the hyperbolic-like isofrequency shapes, so-called *hyperbolic metamaterials*, unlock novel opportunitites for applications such as enhanced emission, planar hyperlensing, high-directional wave propagation, and near-field energy transfer^2,7–9^. *Hyperbolic metasurfaces*, which are ultrathin (quasi-2D) analogues of hyperbolic metamaterials, offer even more practical benefits, enabling unprecedented control over light-matter interactions at the nanoscale^10–12^. Hyperbolic metasurfaces also allow to manipulate light in the near field with remarkable efficiency, e.g., by implementing planar hyperlensing^10,13^, high-directional plasmon excitation^14–16^ and in-plane surface-wave canalization^17–19^. The in-plane control of the hyperbolic plasmon-polaritons in terms of their directivity pattern, localization degree and wavefront shapes is of particular interest^10,11^.

All these applications of hyperbolic metasurfaces require the flexible adjustment of the zeros and poles of the surface conductivity. It is important to control both the spectral positions and amplitudes of the corresponding resonances, the spectral positions of near-zero regions and the ratio of the surface conductivity tensor components. The recent works show the possibilities to engineer the resonances of hyperbolic metasurfaces via the intrinsic geometric anisotropy of meta-atoms^20,21^. In addition to the resonant control of the surface conductivity, recent studies have demonstrated photonic topological flat-band regimes in metasurfaces^22,23^, including ultrahigh-Q quasi-bound states in the continuum^24,25^, associated with lattice-induced flat dispersion. Such flat-band resonances provide an alternative route to extreme field confinement and narrow spectral features, complementing the broadband hyperbolic response explored in this work. However, the engineering of hyperbolic metasurfaces could be also done with even isotropic meta-atoms by introducing the extrinsic structural anisotropy of the meta-atoms’ arrays, which is poorly studied to date.

In this work, we systematically compare two practically relevant routes to break the in-plane symmetry of plasmonic metasurfaces based on the gold nanopatches, such as stretching the meta-atoms and stretching the lattice. By varying the degree of anisotropy (stretching), the period of the metasurface, and the side of the nanopatches, we investigate the spectral positions and amplitudes of the resonances of the effective surface conductivity tensor describing the metasurface properties. We study the emergence of hyperbolicity, plasmon canalization, and near-field hot-spots for both approaches. By tracking the splitting and amplitudes ratio of resonances, we identify an extreme-anisotropy regime, induced by the near-field capacitive coupling in the gap between neighboring nanopatches, whereas the hyperbolic window and canalization become strongly enhanced. Finally, we provide the comprehensive comparison between physical mechanisms and related properties aimed to choose the optimum strategy for the engineering of anisotropic plasmonic resonant metasurfaces depending on the targeted functionality and fabrication facilities. The obtained results are of practical benefits to optical and photonic engineers in a plethora of applications including in-plane signal transferring, optical biosensing, emission enhancement, high-resolution imaging and near-field light control.

## Model and definitions

**Fig. 1:**
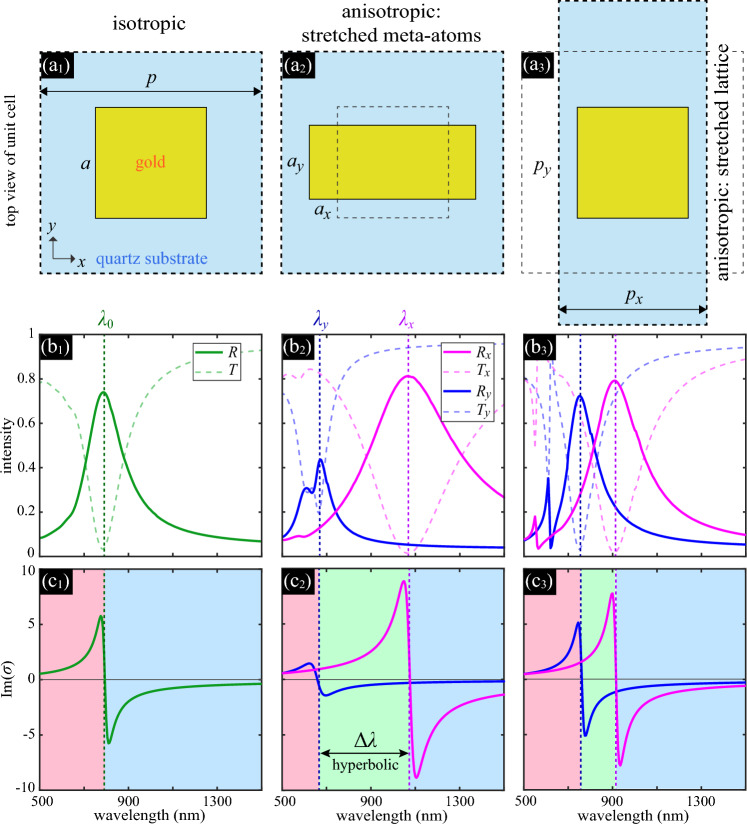
(**a**) The unit cells of (**a1**) isotropic and (**a2**), (**a3**) anisotropic metasurfaces representing gold nanopatches on quartz substrate. (**a1**) Isotropic metasurface: square nanopatch with a side $a = 150$ nm in a periodic square lattice of $p = 300$ nm. (**a2**), (**a3**) Anisotropic metasurfaces formed by the stretching of (a$_2$) nanopatch along *x*-direction and (**a3**) lattice constant along *y*-direction by the factor of $\eta = 1.5$: (**a2**) $a_x = a \, \eta$, $a_y = a/\eta$, (a$_3$) $p_x = p/\eta$, $p_y = p \, \eta$. The initial area of (**a2**) nanopatch and (**a3**) unit cell before stretching is shown by grey dashed lines. (**b**) The corresponding reflectance (solid lines) and transmittance (dashed lines) spectra for (**b1**) isotropic and (**b2**), (**b3**) anisotropic metasurfaces shown in (**a**). The purple and blue lines in (**b2**), (**b3**) correspond to the incident electric field orientation along *x* and *y* axes, respectively. (**c**) The imaginary parts of retrieved surface conductivity spectra, obtained via the fitting of Eq. (5) using the reflectance data in (**b**). The blue, red and green regions correspond to (i) inductive $\left[ \text {Im}\left( \sigma _x \right)> 0, \text {Im}\left( \sigma _y \right) > 0 \right]$, (ii) capacitive $\left[ \text {Im}\left( \sigma _x \right)< 0, \text {Im}\left( \sigma _y \right) < 0 \right]$ and (iii) hyperbolic $\left[ \text {Im}\left( \sigma _x \right) \text {Im}\left( \sigma _y \right) < 0 \right]$ regimes, respectively. The resonant wavelengths for each cases are shown in (**b**) and (**c**) by vertical dashed lines.

**Fig. 2:**
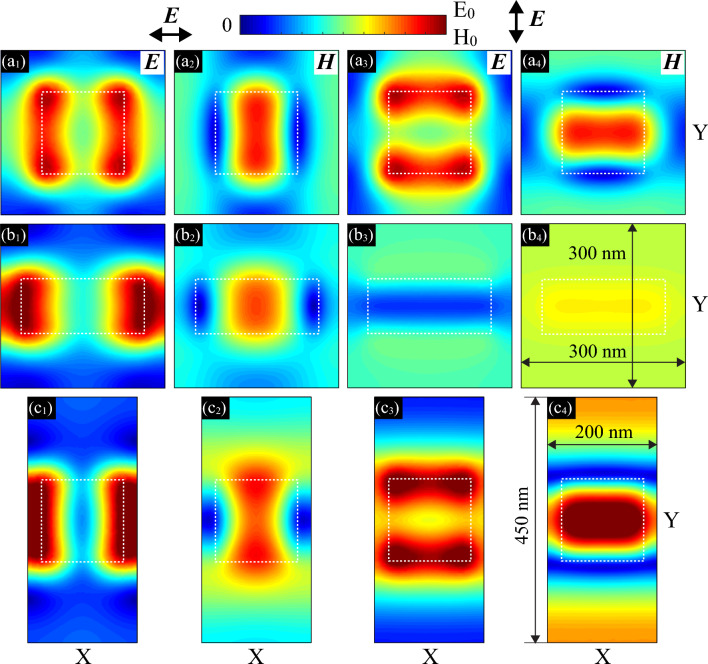
Spatial distributions of the scattered (**a1**), (**b1**), (**c1**), (**a3**), (**b3**), (**c3**) electric and (**a2**), (**b2**), (**c2**), (**a4**), (**b4**), (**c4**) magnetic field amplitudes in *xy*-plane at the distance of 30 nm from a metasurface within an unit cell for (**a1**)–(**a4**) isotropic metasurface, (**b1**)–(**b4**) anisotropic metasurface (stretched meta-atom) and (**c1**)–(**c4**) anisotropic metasurface (stretched lattice) for the incident electric field orientation along (**a1**), (**a2**), (**b1**), (**b2**), (**c1**), (**c2**) *x*-axis and (**a3**), (**a4**), (**b3**), (**b4**), (**c3**), (**c4**) *y*-axis. The field distributions are given at the resonant wavelengths (**a1**)–(**a4**) $\lambda _0$ for isotropic metasurface, (**b1**), (**b2**), (**c1**), (**c2**) $\lambda _x$ and (**b3**), (**b4**), (**c3**), (**c4**) $\lambda _y$ for anisotropic metasurfaces. The white dashed lines show the locations of meta-atoms.

**Fig. 3:**
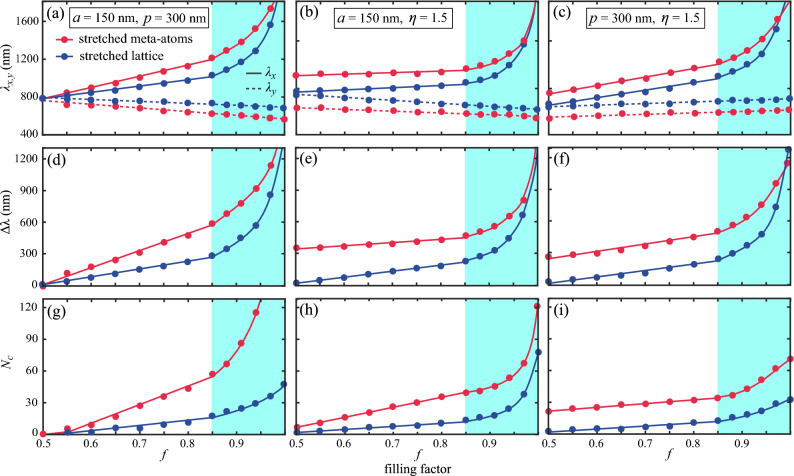
The dependencies of (**a**–**c**) the resonant wavelengths $\lambda _{x}$ (upper solid lines) and $\lambda _{y}$ (lower dashed lines), (**d**–**f**) the spectral difference between the resonances $\Delta \lambda$ for nanoparticle (red lines) and unit cell (blue lines) stretching, and (**g**, **h**) canalization efficiency along *x*-direction $N_{c}$ on the filling factor along the stretching direction *f* in the range from $f = 0.5$ to $f = 0.99$ (extremely anisotropic case) for the fixed (**a**, **d**, **g**) side of a square nanopatch ($a=150$ nm) and period ($p=300$ nm), (**b**, **e**, **h**) side of a square nanopatch ($a=150$ nm) and stretching factor ($\eta = 1.5$), and (**c**, **f**, **i**) period ($p=300$ nm) and stretching factor ($\eta = 1.5$). The cyan regions correspond to the values of $f > 0.85$. Here, the dots correspond to the numerical calculations while the lines to their fitting with linear and quadratic curves for *f* range from 0.5 to 0.85 and from 0.85 to 0.99, respectively.

**Fig. 4:**
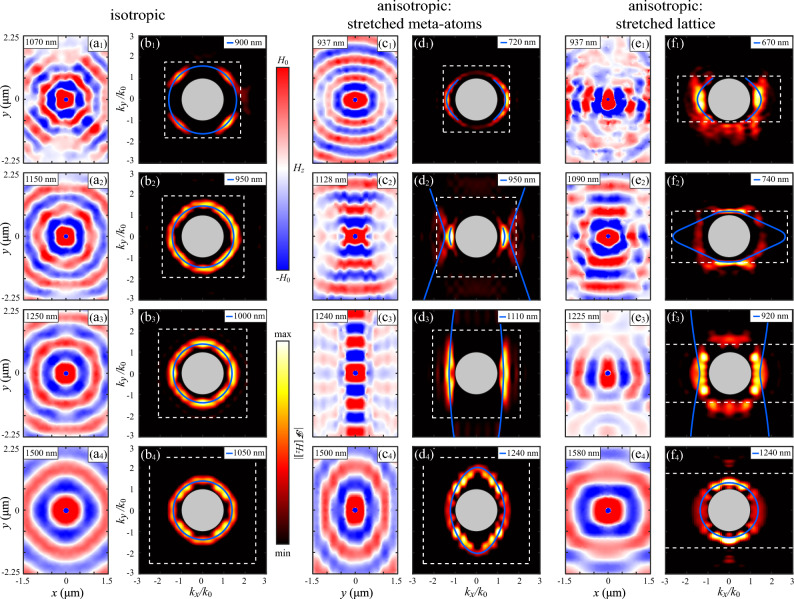
(**a1**)–(**a4**), (**c1**)–(**c4**), (**e1**)–(**e4**) The spatial distributions of the normal components of magnetic field in the plane of a metasurface and (**b1**)–(**b4**), (**d1**)–(**d4**), (**f1**)–(**f4**) the related isofrequency contours of surface waves excited at (**a1**)–(**a4**), (**b1**)–(**b4**) isotropic metasurface, (**c1**)–(**c4**), (**d1**)–(**d4**) anisotropic metasurface (stretched meta-atoms) and (**e1**)–(**e4**), (**f1**)–(**f4**) anisotropic metasurface (stretched lattice) for different wavelengths. The field profiles were calculated for a metasurface consisting of 17$\times$17 unit cells. The isofrequency contours were calculated analytically by solving the dispersion equation of surface waves (solid blue lines) and numerically by the extraction from the field profiles by Fourier transformation (color maps). The grey circles and white dashed lines at isofrequency contours show the light lines in vacuum and the first Brillouin zone, respectively.

We examine the metasurfaces representing the periodic arrays of gold nanopatches with a thickness of $h = 20$ nm, positioned at the interface of a quartz substrate ($n_s = 1.45$). The dispersion of gold is taken from the experimental data for the thin films ^26^. First, we consider the isotropic case, where square nanopatches with a side *a* are packed in a square lattice with a period *p*. This metasurface can be described by the surface conductivity obeying Drude-Lorentz model ^20,27^:

2 $$\begin{aligned} \sigma = \dfrac{i \sigma _0}{\beta + i \gamma }, \end{aligned}$$

where $\sigma _0$ is the amplitude of surface conductivity, $\beta = 1 - \Omega ^2/\omega ^2$ and $\gamma = \Gamma /\omega$ are the dimensionless frequency-dependent resonance- and decay-associated variables, $\Omega$ is the resonant frequency, $\Gamma$ is the resonant bandwidth, $\omega = 2 \pi c / \lambda$ is the angular frequency, *c* is the speed of light.

Then, we switch from the initial isotropic metasurface to anisotropic metasurfaces by stretching either nanopatches or lattice by a stretching factor $\eta$. These metasurfaces can be characterized within the local effective medium approximation by the two-dimensional anisotropic surface conductivity tensor^27–29^:

3 $$\begin{aligned} \hat{\sigma } = \begin{pmatrix} \sigma _x & 0\\ 0 & \sigma _y \end{pmatrix}, \end{aligned}$$

where the tensor components $\sigma _x$ and $\sigma _y$ obey the resonant Drude-Lorentz behavior according to Eq. (2). As a result, we investigate three general cases:

1. Isotropic metasurface: square nanopatches $(a \times a)$ in a square lattice $(p \times p)$, see Fig. 1a$_1$.
2. Anisotropic metasurface (stretched nanoparticles): rectangular nanopatches $(a_x \times a_y)$ in a square lattice $(p \times p)$, see Fig. 1a$_2$. Here, $a_x = a \eta$ and $a_y = a / \eta$.
3. Anisotropic metasurface (stretched lattice): square nanopatches $(a \times a)$ in a rectangular lattice $(p_x \times p_y)$, see Fig. 1a$_3$. Here, $p_x = p / \eta$ and $p_y = p \eta$.

Please note that the initial areas of both the nanopatches and unit cells remain the same after the stretching, namely, $a_x a_y = a^2$ and $p_x p_y = p^2$. The switch from isotropic to anisotropic metasurfaces in this case is accompanied by the breaking the in-plane $C_{4v}$ symmetry of the isotropic metasurface down to $C_{2v}$ one.

As an illustrative example, we consider an isotropic metasurface with a lattice constant of $p = 300$ nm consisting of a square nanopatches with a side of $a = 150$ nm [Fig. 1a$_1$]. Then, we stretch nanopatches along *x*-axis [Fig. 1a$_2$] or unit cells along the *y*-axis [Fig. 1a$_3$] transforming to the anisotropic metasurfaces described by Eq. (3). We calculate the reflectance and transmittance spectra for each type of metasurfaces, see Fig. 1b$_1$–b$_3$. By fitting the reflectance spectra we extract the effective surface conductivities for each case, as it is shown in Fig. 1c$_1$–c$_3$. The details of the numerical calculation of spectra and the retrieval procedure of the surface conductivity tensor can be found in Methods section.

The resonant wavelengths are defined as $\lambda _0$ for the isotropic metasurface, $\lambda _x$ and $\lambda _y$ for anisotropic metasurfaces. To conduct further analysis of anisotropic metasurfaces, we introduce the spectral difference between the resonances (SDBR) defined as the difference between the resonant wavelengths along the main axes of surface conductivity tensor $\Delta \lambda = \lambda _x - \lambda _y$ [marked by the green areas in Fig. 1c$_2$–c$_3$]. The imaginary parts of the surface conductivity tensor components within this zone have different signs, which is an inherent feature of the hyperbolic regime within local effective medium approximation:

4 $$\begin{aligned} \text {Im}\left( \sigma _x \right) \text {Im}\left( \sigma _y \right) < 0. \end{aligned}$$

In other words, SDBR defines the spectral width of the hyperbolic regime, emerging between resonances in the wavelength range $\lambda _y< \lambda _0 < \lambda _x$ and characterizing by the condition (4).

We also introduce a filling factor along the *x*-direction, defined as $f = a_x/p_x$. In our case, $f = 0.5$ for isotropic metasurface. It is worth noting the extremely anisotropic case of stretching ($f \approx 1$), when the nanopatches transform to gratings at $a_x \approx p_x$. In this case, the filling factor *f* is proportional to the anisotropy degree defined by the stretching factor $\eta$. Finally, in order to define the degree of divergence in the canalization regime for a specific hyperbolic metasurface design, we determine the ratio between the surface conductivity tensor components (SCTC) $N_{c} = \text {max} \left[ {\text {Im}\left( \sigma _x \right) }/{\text {Im}\left( \sigma _y \right) } \right]$, corresponding to the efficiency of surface plasmons propagation in two mutually orthogonal directions.

## Results

The resonant wavelength $\lambda _0 = 790$ nm of isotropic metasurface splits into two resonances $\lambda _x > \lambda _0$ and $\lambda _y < \lambda _0$ equal to 1070 nm, 660 nm and 920 nm, 760 nm for stretched meta-atoms and stretched lattice, respectively, see Fig. 1c$_1$–c$_3$. It is associated with the breaking of the TE-TM degeneracy of the eigenmodes by the anisotropy. The coincident resonances of pure TE and TM eigenmodes ($\lambda _0$) split into the resonance of quasi-TM ($\lambda _x$) and quasi-TE ($\lambda _y$) modes ^28^. One can notice that $\Delta \lambda$ is almost twice larger for the stretched meta-atoms, than for the stretched lattice. The resonance at $\lambda _x$ is larger for the stretched meta-atom, the opposite situation takes place for $\lambda _y$.

The stretching of the metasurface leads to the redistribution of the electromagnetic fields and the formation of the near-field hot-spots. The scattered field distributions at the resonant wavelengths for all three cases in *xy*-plane at the distance 30 nm in the quartz from the edge of nanodisks are shown in Fig. 2. For isotropic metasurface, the electric field is localized near the edges of meta-atoms, while the magnetic field is concentrated near the centers of nanopatches, both fields are oscillating along the orientation of the incident electric field, see Fig. 2a$_1$–a$_4$. By stretching the meta-atom, the intensity of the electric field in the hot-spots increases along the stretching direction [Fig. 2b$_1$] and decreases across the orthogonal one [Fig. 2b$_3$] highlighting the difference in the resonances of the surface conductivity tensor components shown in Fig. 1c$_2$. For the anisotropic metasurface formed by the lattice stretching, the amplitudes of the resonances and hot-spots along *x*- and *y*-directions are comparable [Fig. 2c$_1$–c$_4$]. The strongest magnetic field is observed for the lattice stretching, when the field is oriented across the direction of the stretching [Fig. 2c$_4$].

We analyze the spectral positions of resonances ($\lambda _x$ and $\lambda _y$), SDBR ($\Delta \lambda$) and SCTC ratio ($N_c$) by stretching nanopatches and unit cells from the isotropic case ($p = 300$ nm, $a = 150$ nm, $f = 0.5$) up to the extremely anisotropic one characterized by *f* = 0.99 (Fig. 3). First, we fix the nanopatches’ size as $a = 150$ nm and lattice constant as $p = 300$ nm, and change the stretching factor $\eta$ from 1 (square nanopatch in square lattice) to 1.98 (almost linear grating), namely, to the rectangular nanopatch with sizes $a_x = 297$ nm, $a_y = 76$ nm and to the rectangular unit cell with sizes $p_x = 151.5$ nm and $p_y = 594$ nm [Fig. 3a,d,g]. The stretching of the nanopatches and lattice are shown by the red and blue lines, respectively, in Fig. 3. Second, we fix the nanopatches’ size as $a = 150$ nm and stretching factor as $\eta = 1.5$, and change the period *p* from 450 to 227.3 nm [Fig. 3b, e, h]. For the case of stretched nanopatches, we fix the nanopatch as $a_x = 225$ nm, $a_y = 100$ nm and vary the period, while for the case of stretched lattice, the unit cell sizes vary from $p_x = 151.5$ nm, $p_y = 341$ nm to $p_x = 300$ nm, $p_y = 675$ nm. Third, we fix the period as $p = 300$ nm and stretching factor as $\eta = 1.5$, and change the nanopatches’ size *a* from 100 to 198 nm [Fig. 3c, f, i]. For the case of stretched lattice, we fix the unit cell sizes as $p_x = 200$ nm, $p_y = 450$ nm and vary the size of a square nanopatch, while for the case of stretched nanopatches, the sizes of meta-atom vary from $a_x = 150$ nm, $a_y = 66.7$ nm to $a_x = 297$ nm, $a_y = 132$ nm.

One can notice that the resonant wavelength $\lambda _y$ linearly blueshifts and redshifts for first/second and third cases, respectively, as the stretching increases for any degree of anisotropy. The resonant wavelength $\lambda _x$ increases linearly up to $f \approx 0.85$ and then non-linearly for $f > 0.85$ for both types of induced anisotropy. For high filling factors, the inter-particle gap $g = p_x - a_x$ becomes sufficiently small, and the coupling mechanism transitions from dipolar lattice interaction to strong near-field capacitive coupling between adjacent nanopatches. In this regime, the response is governed by a gap-induced capacitance $C_{\textrm{gap}} \propto 1/g$, leading to a strongly nonlinear dependence of the resonant wavelength on *g* and, consequently, on *f* (see Supplementary Material, Section S2). We define the case $f > 0.85$ as the extreme anisotropy characterized by the sharp increase of the wavelength of the amplitude-dominant resonance with further increasing of anisotropy. As a consequence, the SDBR ($\Delta \lambda$) and SCTC ratio ($N_c$) also have the corresponding linear and non-linear dependence on the anisotropy for $f \le 0.85$ and $f > 0.85$, respectively. Therefore, one should engineer the metasurfaces with extreme anisotropy to obtain the spectrally broadband hyperbolicity.

For the first case of varying stretching factor, the SDBR varies linearly from 0 to 295 nm and 615 nm at $f = 0.85$, and then non-linearly to 865 nm and 1140 nm at $f = 0.97$ for the stretched lattice and nanopatches, respectively [Fig. 3d]. For the second case of varying period, the SDBR varies linearly from 20 nm and 360 nm at $f = 0.5$ to 235 nm and 480 nm at $f = 0.85$, and then to 670 nm and 800 nm at $f = 0.97$ for the stretched lattice and nanopatches, respectively [Fig. 3e]. For the third case of varying nanopatch size, the SDBR varies linearly from 35 nm and 270 nm at $f = 0.5$ to 260 nm and 520 nm at $f = 0.85$, and then to 740 nm and 970 nm at $f = 0.97$ for the stretched lattice and nanopatches, respectively [Fig. 3f]. Therefore, one should engineer the metasurfaces with extreme anisotropy to obtain the spectrally broadband hyperbolicity. In the extreme anisotropy regime, the stretching of lattice and nanopatches quickly converge to the same results as far as geometry approaches the grating. The SCTC ratio also has the similar behavior reaching approximately from 12 to 18 and from 35 to 55 at $f = 0.85$ for different cases of the stretched lattice and nanopatches, respectively [Fig. 3g–i]. However, stretching the unit cell can achieve a maximum of $N_c \approx 35$ even in the extreme anisotropy regime up to $f < 0.97$, while the corresponding values of the SCTC ratio are at least twice higher for the case of stretched nanopatches.

The effective surface conductivity tensor obtained can be used to study the near-field properties of hyperbolic metasurfaces. Specifically, one can solve the dispersion equations for surface waves and study the topology of the isofrequency contours for each case. It could be done analytically within the local medium approximation using the effective surface conductivity tensor extracted from the reflectance spectra and shown in Fig. 1c$_1$–c$_3$. The isofrequency contours derived analytically in the approximation of symmetric environment with the surrounding permittivity of 1.5 (average between the refractive indices of substrate and superstrate) are shown by the solid lines in Fig. 4. To verify these results and to evaluate the impact of the spatial dispersion, we conduct the full-wave numerical simulation of the electromagnetic fields of surface waves excited at hyperbolic metasurfaces. The spatial distributions of the normal component of magnetic field were calculated in the plane of metasurface at the distance 80 nm from the top edge of the gold nanopatches. The surface waves were excited by the loop-like magnetic dipole located in the center of full-scale metasurface consisting of 17$\times$17 unit cells. By applying the two-dimensional Fourier transform to the calculated field profiles, we extract the corresponding isofrequency contours shown by the color maps in Fig. 4. While the topological behaviors of the isofrequency contours, derived semi-analytically using the effective surface conductivity and retrieved numerically from the field profiles, are similar, the proper wavelengths are shifted by approximately 20%, 17% and 27% for isotropic metasurface, anisotropic metasurface with stretched meta-atoms and anisotropic metasurface with stretched lattice, respectively. This discrepancy is primarily associated with spatial dispersion effects. Near the resonances, where the in-plane wavevector approaches the Brillouin zone boundary, the nonlocal correction leads to a relative shift on the order of 20-30%, consistent with the observed deviation (see n Material, Section S1). The effect is most pronounced for the stretched lattice, where spatial dispersion is enhanced due to stronger inter-cell coupling. The numerical calculation in the time domain and the simplification of the dispersion equation to the symmetric case also impact on this discrepancy. The details of the numerical simulation and the isofrequency reconstruction are given in Methods section.

For an isotropic metasurface, surface waves diverge radially from the excitation source at approximately the same group velocity in all directions [Fig. 4a$_1$–a$_4$]. The diameter of the central blue circle in the in-plane distribution is proportional to the distance from the excitation dipole location. In isofrequency contours, this type of wave propagation corresponds to circles with larger radii than light cones [Fig. 4b$_2$–g4b$_4$]. As the wavelength decreases, the circle becomes more deformed due to the high localization and the approach of the isofrequency contour to the boundaries of the first Brillouin zone [Fig. 4b$_1$]. For anisotropic metasurfaces, the isofrequency contour elongates along and across the stretching direction for wavelengths smaller ($\lambda < \lambda _y$) and larger ($\lambda > \lambda _x$) than the resonant wavelengths, respectively, thus transforming from a circle into an ellipse [Fig. 4d$_1$, d$_4$, f$_1$ and f$_4$]. It is accompanied by the corresponding axial-asymmetric diverging field profiles, which inherit the symmetry of the isofrequency contours [Fig. 4c$_1$, c$_4$, e$_1$ and e$_4$]. The plasmon canalization regimes are observed in the field profiles in Fig. 4c$_3$ and e$_2$–e$_3$. The corresponding isofrequency contours represent two nearly parallel lines (near-flat isofrequency contour), see Fig. 4d$_3$ and f$_2$–f$_3$. The surface wave is excited the most efficiently along the directions with smaller values of wavevector defining the direction of plasmon canalization perpendicular to the near-parallel lines at the isofrequency contours. The most pronounced canalized surface-wave propagation is observed for the anisotropic metasurface formed by the meta-atoms stretching [Fig. 4c$_3$], which can be characterized as the extreme canalization.^19^. At the same time, for the anisotropic metasurface formed by the lattice stretching there are two mutually orthogonal directions of plasmon canalization in the vicinity of $\lambda _x$ and $\lambda _y$, respectively. The hyperbolic-like field profiles and isofrequency contours can be noticed for the anisotropic metasurface with stretched meta-atoms in Fig. 4d$_2$. So, one can clearly notice the photonic topological transitions of isofrequency contours as they change from an ellipse elongated across the stretching direction (closed loop) [Fig. 4d$_1$ and e$_1$] to hyperbolas and near-flat contour (open loops) [Fig. 4d$_2$–d$_3$ and e$_2$–e$_3$], and then back to an ellipse elongated along the stretching direction (closed loop) [Fig. 4d$_4$ and e$_4$] as the wavelength increases.

## Discussion

We have shown that introducing in-plane anisotropy by geometric stretching for a metasurface creates an anisotropic platform that supports a hyperbolic spectral window, plasmon canalization and pronounced near-field hot-spots. The photonic topological transitions of the isofrequency contours directly links the properties of metasurface and its engineering procedure. In addition, we identified the regime of extreme anisotropy, where the amplitude and spectral positions of the surface conductivity tensor resonances increases sharply, enabling the strongest canalization and broadband hyperbolicity. Although both meta-atom versus lattice stretching procedures reduce the in-plane symmetry from $C_{4v}$ to $C_{2v}$, the resulting resonance splitting and near-field hot-spot formations are governed by different physical mechanisms. In the case of stretched meta-atoms, the anisotropy is introduced directly through the geometry of the individual nanopatch, which modifies its polarizability along the principal axes and leads to a strong splitting of the quasi-TM and quasi-TE resonances. In contrast, for stretched lattices the nanopatches remain individually isotropic, and the splitting originates predominantly from anisotropic collective coupling between neighboring resonators along the two lattice directions. The lattice-induced correction remains weaker than the direct anisotropy of the resonator shape for subwavelength periods, and the corresponding resonance splitting is smaller.

These results provide a direct guideline for choosing the anisotropic metasurface engineering strategy depending on the targeted functionality and fabrication methodology. To achieve broadband hyperbolicity and extreme canalization, anisotropic metasurfaces based on stretched meta-atoms are preferable, since reshaping the resonator boosts the resonance splitting and yields higher anisotropy contrast. This route is also advantageous when strong electric hot-spots and maximal near-field enhancement are required. In contrast, for frequency-tunable functionalities and switchable plasmon canalization directions, anisotropic metasurfaces based on a stretched lattice offer a more suitable route, because the anisotropy is primarily encoded in the lattice inter-coupling and can support canalization along different axes at different wavelengths. Stretching the meta-atoms is generally more robust to positional disorder because the dominant contribution is governed by the individual resonator response. However, it is more sensitive to fabrication imperfections in the meta-atom geometry (e.g., aspect-ratio errors, thickness deviations, rounding, edge roughness, etc.). Conversely, stretching the lattice is typically less sensitive to resonator-shape imperfections, since the response is more strongly influenced by collective lattice effects, but it can be more vulnerable to disorder in the array periodicity and to non-local effects. The correspondence between the attainable properties and the anisotropy-inducing approaches is summarized in Table 1.

The proper engineering of the surface conductivity of anisotropic metasurfaces is also important for far-field applications, such as anomalous refraction, perfect absorption and Brewster’s effect^30–32^. The adjustment of the interplay between quasi-TE and quasi-TM surface waves at hyperbolic metasurfaces could be useful for spin-orbit interactions of light^33–36^ and chiral sensing^37–40^. Another appealing directions include Moiré photonics and twisted optics requiring the engineering of multilayered systems of plasmonic hyperbolic metasurfaces^41–44^. Please note that plasmon canalization in optical and microwave hyperbolic metasurfaces is governed by the same mechanism - the flattening of the isofrequency contour enabling diffractionless propagation. However, in optics it originates from intrinsic plasmonic resonances of nanoparticles, whereas in microwaves it arises from spoof plasmons supported by patterned metallic structures with effective LC resonances^19,44,45^.

**Table 1:** Comparison of the different properties of the three types of metasurface studied in this work.

Properties	Isotropic	Anisotropic (stretched meta-atom)	Anisotropic (stretched lattice)
Resonant wavelengths	1	2	2
Amplitudes of resonances	Equal	Strongly different	Different, but comparable
Dominant resonant mechanism	Balanced	Plasmon resonance of meta-atom	Collective lattice effects
Number of topological transitions	0	2	2
Width of hyperbolic regime	Absent	Wide	Narrow
Plasmon canalization	Absent	Yes, extreme	Yes
Canalization direction	Absent	1	2
Modes hybridization	No	Weak	Strong
Electric hot spots	Medium	Strong	Medium
Magnetic hot spots	Medium	Medium	Strong
Spatial dispersion	Weak	Weak	Strong
Frequency-tunable	Independent	Robust	Selective
Disorder impact	Medium	Weak	Strong
Fabrication imperfections impact	Medium	Strong	Weak

## Methods

*Numerical simulation of reflectance/transmittance spectra and scattered fields* We calculated the reflectance and transmittance spectra for the hyperbolic metasurfaces based on the finite-element method using the Frequency Domain in COMSOL Multiphysics software. We consider the excitation of a plane wave under normal incidence by a periodic port for a single unit cell of a metasurface, applying periodic Floquet boundary conditions at side walls of the box. The upper and lower sides were substituted by the perfect matched layers. The calculated reflectance and transmitted spectra are shown in Fig. 1b$_1$–b$_3$. The related spatial distributions of the scattered fields for this configuration are shown in Fig. 2.

*Retrieval procedure of effective surface conductivity tensor* The Fresnel equations with a 2D conducting sheet connect the reflectance and surface conductivity. We assume the surface conductivity obeys Drude-Lorentz model according to Eq. (2). The extraction of the effective surface conductivity has been done by fitting the reflectance spectra, shown in Fig. 1b$_1$–b$_3$, with a formula^20,21^:

5 $$\begin{aligned} R_i = \dfrac{(\sigma _0^i)^2+(\beta _i^2+\gamma _i^2) \zeta _{-}^2 + 2\gamma _i \sigma ^i_0 \zeta _{-}}{(\sigma ^i_0)^2+(\beta _i^2+\gamma _i^2)\zeta _{+}^2 + 2\gamma _i \sigma _0^i \zeta _{+}}, \end{aligned}$$

where the index *i* corresponds to *x* and *y* components, and $\zeta _{+,-} = n_s \pm 1$. Fitting the simulated spectra of reflectance by the least squares method, we retrieve the $\sigma _0$, $\beta$ and $\gamma$ parameters of surface conductivity. The retrieved surface conductivities for different types of hyperbolic metasurfaces are shown in Fig. 1c$_1$–c$_3$. The calculated data was also used for the analysis done in Fig. 3.

*Calculation of isofrequency contours via effective surface conductivity tensor* The calculation of isofrequency contours have been done using the extracted surface conductivity by solving the dispersion equation for hyperbolic plasmon-polaritons in a symmetric case^27,28^:

6 $$\begin{aligned} \left( \dfrac{2 \varepsilon k_0}{\kappa } + i \sigma _{xx} \right) \left( \dfrac{2 \kappa }{\mu k_0} - i \sigma _{yy} \right) = \sigma _{xy} \sigma _{yx}, \end{aligned}$$

where $\kappa = \sqrt{k^2_{\text {HPP}} - \varepsilon \mu k_0^2}$ is the penetration depth, $k_{\text {HPP}}$ is the in-plane component of the hyperbolic plasmon-polariton wavevector, $\varepsilon$ ($\mu$) is permittivity (permeability) of the surrounding medium, $\sigma _{ij}$ are the components of the surface conductivity tensor in the rotated coordinate system defined as $\sigma _{xx,yy} = \sigma _{x,y} \cos ^2{\alpha } + \sigma _{y,x} \sin ^2{\alpha }$ and $\sigma _{xy,yx} = (\sigma _x - \sigma _y) \cos {\alpha } \sin {\alpha }$, $\alpha$ is the angle between the *x*-axis and the propagation direction of the HPP. Here, we consider a metasurface surrounded by an isotropic medium with permittivity $\varepsilon = [(n_s + 1)/2]^2$. The isofrequency contours calculated analytically using the retrieved effective surface conductivity tensor are shown by the solid lines in Fig. 4.

*Numerical simulation of spatial distributions of electromagnetic fields* We carry out numerical modeling of the electromagnetic field distribution of the full-scale hyperbolic metasurfaces consisting of 17 $\times$ 17 unit cells using the Time Domain Solver in CST Studio Suite. Open boundary conditions are applied to all sides of the simulation domain to prevent artificial reflections and to emulate free-space propagation. The surface waves were excited by the electric-like or magnetic-like dipole located in the center of a metasurface at the distance 100 nm from a metasurface^19^. The calculated field profiles have been exported with a fine sampling of 20 nm, which provides sufficient resolution to analyze the near-field structure. The spatial distributions of the normal components of electric and magnetic fields for isotropic and anisotropic metasurfaces are shown in Fig. 4.

*Extracting isofrequency contours from calculated field profiles* By applying the two-dimensional Fourier transform to the numerically calculated field profiles, we retrieved the corresponding isofrequency contours as the color maps^19^. This approach allows the eigenmodes to be mapped out as the peaks of the intensity in a color map corresponding to the wavevector components presented in the field profiles calculations. The isofrequency contours extracted from the numerically calculated field profiles are shown by the color maps in Fig. 4.

## Supplementary Information

Supplementary Information.

## Data Availability

All data supporting the findings of this study are available within the paper .
