# Supplementary material for: Photonic topological transitions for surface waves with resonant plasmonic metasurfaces: interplay between meta-atom and lattice stretching
Source: Sci Rep. 2026 May 7;16:20619. doi: 10.1038/s41598-026-50214-w (PMC13333844; doi:10.1038/s41598-026-50214-w)
Supplement: Supplementary file 1 — Supplementary Information. [file 41598_2026_50214_MOESM1_ESM.pdf]

Supplementary Material for "Photonic topological transitions for surface waves with resonant plasmonic metasurfaces: interplay between meta-atom and lattice stretching"

Artem Hrinchenko[1,*], Veronika Batianova[2], Sergey Polevoy[3], and Oleh Yermakov[4,*]

[1]V. N. Karazin Kharkiv National University, Kharkiv, Ukraine
[2]Abbe School of Photonics, Friedrich Schiller University, Jena, Germany
[3]Department of Radiospectroscopy, O. Ya. Usikov Institute for Radiophysics and Electronics of the NASU, Kharkiv, Ukraine
[4]Department of Fiber Photonics, Leibniz Institute of Photonic Technology, Jena, Germany
[*]a.hrinchenko@karazin.ua
[*]oleh.yermakov@leibniz-ipht.de

ABSTRACT

Here, we discuss the impact of spatial dispersion on the surface plasmon dispersion and resonant wavelengths (S1) and the analytical model of extreme anisotropy (S2).

S1 Impact of spatial dispersion on surface plasmon dispersion and resonant wavelengths

The retrieval procedure for the surface conductivity is done within the local effective medium approach. It works well for low filling factors, but the non-local effects can be significant for the specified configurations of the stretched metasurfaces.[1] Here, we derive the simple analytical approximation based on the perturbation theory to evaluate the impact of the spatial dispersion.

To quantify the role of spatial dispersion, we estimate the leading-order nonlocal correction to the surface conductivity in the form:

$$\sigma(\omega, k_{\|}) = \sigma_{\text{eff}} + \sigma_{\text{sp}} p^2 k_{\|}^2, \tag{S1}$$

Within this approach, the total surface conductivity $\sigma(\omega, k_{\|})$ consists of the effective surface conductivity derived with the local effective medium approach[2] (σ_{eff}) and the additional conductivity term proportional to the square of a wavevector, σ_{sp} is the conductivity-related amplitude, p is the period of the metasurface and $k_{\|}$ is the in-plane wavevector of the surface plasmon along the propagation direction. The non-local contribution to the wavevector then is described with $\delta k_{\|}^{\text{sp}}$ as follows:

$$k_{\|} = k_{\|}^0 + \delta k_{\|}^{\text{sp}}, \tag{S2}$$

where $k_{\|}^0$ is the wavevector obtained within local effective medium approach.

By solving Eq. (6) one derives the dimensionless amendment to the effective surface conductivity as:

$$\delta\sigma = \frac{\sigma_{\text{sp}}}{\sigma_{\text{eff}}} = \frac{\delta k_{\|}^{\text{sp}}}{k_{\|}^0} \frac{1}{p^2 \left[(k_{\|}^0)^2 - \varepsilon\mu k_0^2\right]}. \tag{S3}$$

It results in the corresponding spectral shift between the initial wavelength λ_0 and the wavelength taken into account the non-local term λ. The relative dimensionless amendment to the wavelength can be defined as

$$\delta\lambda = \frac{\lambda - \lambda_0}{\lambda}. \tag{S4}$$

The comparison between the local and non-local dispersion curves along different propagation directions are shown in Fig. S1. The dispersions $\omega(k_{\|}^0)$ and $\omega(k_{\|})$ are calculated analytically using the effective surface conductivity approach (see

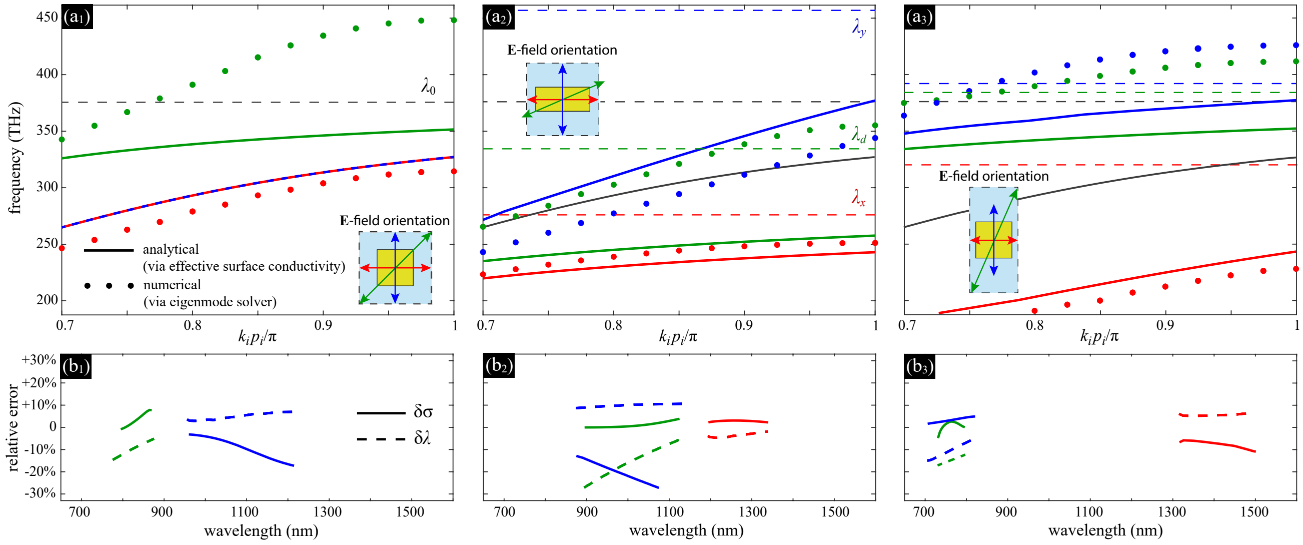

Figure S1. (a) The dispersions of surface plasmons within the first Brillouin zone propagating along x-axis (red), diagonal (green) and y-axis (blue) directions as it is shown in the insets. (a_1) Isotropic metasurface: square nanopatch with a side $a = 150$ nm in a periodic square lattice of $p = 300$ nm. (a_2)-(a_3) Anisotropic metasurfaces formed by the stretching of (a_2) nanopatch along x-direction and (a_3) lattice constant along y-direction by the factor of $\eta = 1.5$. The solid lines and dots correspond the dispersion curves obtained analytically using the effective surface conductivity and numerically using eigenmode analysis. The horizontal dashed lines show resonant wavelengths within the analytical model. The gray solid and dashed lines in (a_2)-(a_3) mark the isotropic case for the comparison. (b) The corresponding relative amendments to the wavelength and surface conductivity calculated according to Eqs. (S3) and (S4) are shown by the dashed and solid lines, respectively.

Cases	$\delta\lambda_x^{\max}$	$\delta\lambda_y^{\max}$	$\delta\lambda_d^{\max}$
Isotropic	4%	4%	27.5%
Anisotropic (stretched meta-atoms)	3.9%	9%	32%
Anisotropic (stretched lattice)	6.2%	14.9%	17%

Table S1. Comparison of the maximum values of the relative amendments to the resonant wavelengths for the three types of metasurface studied in this work and three directions: along x-axis ($\delta\lambda_x^{\max}$), along y-axis ($\delta\lambda_y^{\max}$) and along diagonal direction ($\delta\lambda_d^{\max}$).

Methods section) and numerically using Eigenmode Solver of COMSOL Multiphysics [Fig. S1(a)]. It is intuitively clear that the most non-local contribution is presented for the diagonal directions of propagation. The maximum spatial-dispersion-associated deviation is observed in the vicinity of resonances, where the wavevector achieves its near-maximum values.

The corresponding relative amendment values for the resonant wavelengths and surface conductivity are shown in Fig. S1(b) and summarized in Table S1. The maximum impact of the spatial dispersion along the specific direction is achieved for the stretched meta-atom, while the total non-local impact averaged over all the directions is observed for the stretched lattice. This estimate is consistent with the numerical results shown in Fig. 4, where the deviation reaches up to 20-30% near the resonance. Importantly, the largest discrepancy occurs for propagation directions with large in-plane wavevectors (e.g., diagonal direction).

S2 Analytical toy model of extreme anisotropy

The regime of extreme anisotropy observed for filling factor $f > 0.85$ can be associated with strong near-field interaction between adjacent nanopatches along x-direction when the inter-particle gap

$$g = p_x - a_x = p_x(1 - f) \tag{S5}$$

becomes sufficiently small. In this regime, the electromagnetic coupling can no longer be treated as a weak perturbative lattice interaction and instead acquires a pronounced capacitive character. Physically, the metasurface can be viewed as an

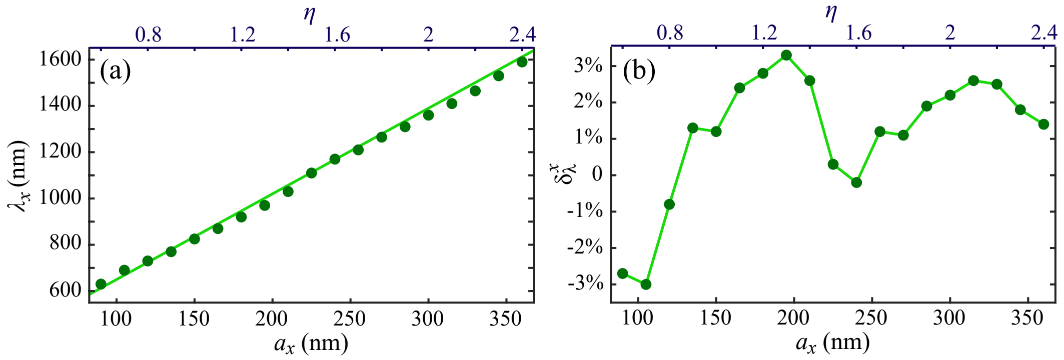

Figure S2. Dependencies of (a) the resonant wavelength calculated numerically (dots) and analytically using the fitting formula (S7) and (b) the corresponding relative error in the resonant wavelength [Eq. (S9)] between fitting formula and numerical calculations on the side of a single isolated nanopatch along the elongated direction a_x and the stretching factor η.

array of coupled plasmonic resonators, whose resonance is governed by an effective LC-response. As the gap decreases, an additional gap capacitance $C_{\rm gap} \propto 1/g$ emerges due to the accumulation of opposite charges at the facing edges of neighboring nanopatches.

The final expression for the resonant wavelength can be expressed as:

$$\lambda^{\rm res} = \lambda_0^{\rm res} + \lambda^{\rm int} + \lambda_g^{\rm res}, \tag{S6}$$

where $\lambda_0^{\rm res}$ is the resonant wavelength of the single isolated nanoparticle (i.e., not embedded in the lattice), $\lambda^{\rm int}$ is the shift in the resonant wavelength associated with lattice coupling between the neighboring nanopatches in the periodic array, and $\lambda_g^{\rm res}$ is the capacitive gap-induced contribution to the resonant wavelength.

In our geometry, the resonant wavelength of the single nanopatch is well approximated by the following linear fitting (see Fig. S2):

$$\lambda_{0,i}^{\rm res} = 3.7a_i + 280 \text{ nm}, \tag{S7}$$

where $i = x, y$. In the first-order approximation, the lattice-coupling-induced shift is assumed to be constant and independent of both the period and the nanopatch size: $\lambda^{\rm int} = C = const$ (nm). The gap-induced term $\lambda_g^{\rm res}$ is proportional to the gap capacitance, so $\lambda_g^{\rm res} = A\sqrt{a}/\sqrt{g}$ (nm). At the same time, the gap-related term is negligibly small for the y-direction (i.e., we assume $A_y = 0$), and amendment interaction wavelength is smaller for y-direction, than for x-direction ($C_y < C_x$). Finally, we obtain the following expressions for the resonant wavelength along $x-$ and $y-$directions for the stretched meta-atoms and lattice:

$$\begin{aligned}
\lambda_{{\rm part},x}^{\rm res} &= \lambda_{0,x}^{\rm res} + C_{{\rm part},x} + \frac{A_{{\rm part},x}\sqrt{a}}{\sqrt{p-a_x}},\\
\lambda_{{\rm part},y}^{\rm res} &= \lambda_{0,y}^{\rm res} + C_{{\rm part},y},\\
\lambda_{{\rm latt},x}^{\rm res} &= \lambda_{0,x}^{\rm res} + C_{{\rm latt},x} + \frac{A_{{\rm latt},x}\sqrt{a}}{\sqrt{p_x-a}},\\
\lambda_{{\rm latt},y}^{\rm res} &= \lambda_{0,y}^{\rm res} + C_{{\rm latt},y}.
\end{aligned} \tag{S8}$$

Please note that gap is defined as $g = p - a_x$ and $g = p_x - a$ for the stretched naoparticle and lattice, respectively.

Figure S3 shows the comparison between the resonant wavelengths fitted with Eq. (S8) and calculated numerically in COMSOL Multiphysics (see Methods section) for three typical cases considered in this work [Figs. S3(a)-S3(c)], and the related differences between the resonant wavelengths $\Delta\lambda$ [Figs. S3(d)-S3(f)]. The corresponding fitting coefficients A and C are summarized in Table S2. The relative errors between numerically calculated and fitted with formula (S8) resonant wavelengths along x-direction, whereas the extreme anisotropy occurs, defined as

$$\delta_\lambda^i = \frac{|\lambda_i^{\rm num} - \lambda_i^{\rm fit}|}{\lambda_i^{\rm num}} \tag{S9}$$

do not extend 10% [Figs. S3(g)-S3(i)].

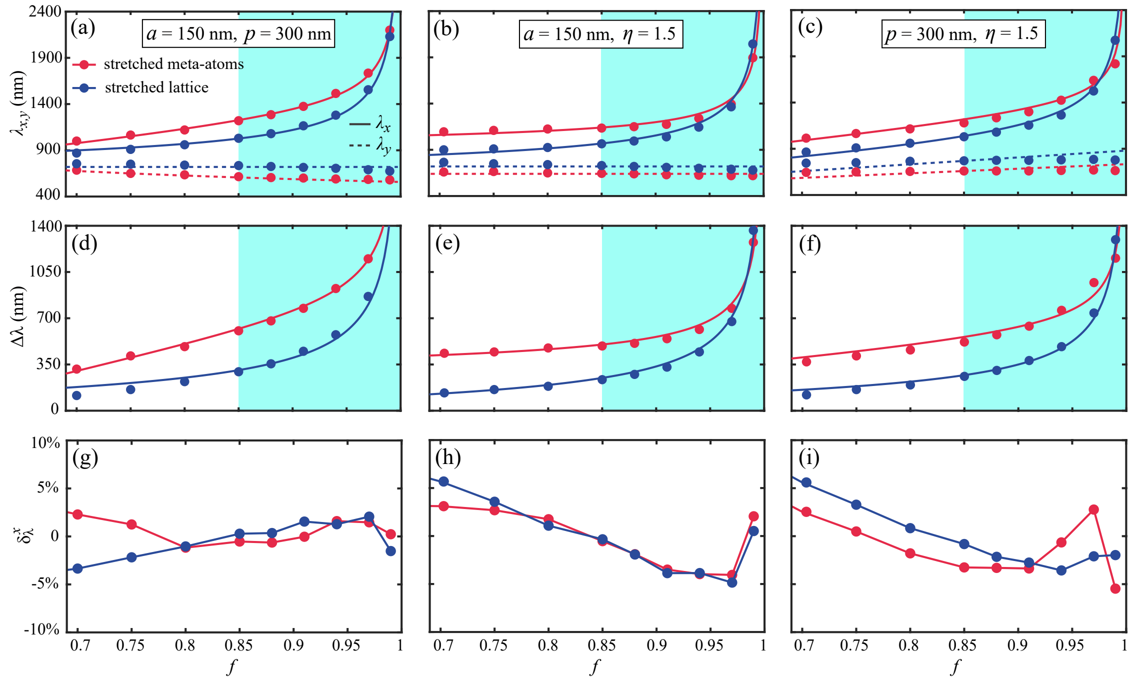

Figure S3. The dependencies of (a-c) the resonant wavelengths λ_x (upper solid lines) and λ_y (lower dashed lines), (d-f) the spectral difference between the resonances $\Delta\lambda$ for nanoparticle (red lines) and unit cell (blue lines) stretching, and (g-h) the corresponding relative error in the resonant wavelength [(S9)] along x-direction on the filling factor along the stretching direction f in the range from $f = 0.5$ to $f = 0.99$ (extremely anisotropic case) for the fixed (a,d,g) side of a square nanopatch ($a = 150$ nm) and period ($p = 300$ nm), (b,e,h) side of a square nanopatch ($a = 150$ nm) and stretching factor ($\eta = 1.5$), and (c,f,i) period ($p = 300$ nm) and stretching factor ($\eta = 1.5$). In (a-f) the cyan regions correspond to the values of $f > 0.85$, the dots correspond to the numerical calculations while the lines to their fitting with formula (S8), respectively.

Thus, the quadratic-like behavior observed in Fig. 3 for $f > 0.85$ should be understood as an effective approximation to this strongly nonlinear dependence within the studied parameter range. Importantly, the sharp behavior change in the vicinity of $f \approx 0.85$ reflects a transition in the dominant physical mechanism: from dipolar lattice-mediated coupling at moderate filling factors to the near-field capacitive interaction between neighboring elements in the extreme anisotropy regime. This also explains why both types of stretching converge to similar behavior as $f \to 1$, since the response becomes primarily governed by the gap rather than by the individual resonator geometry.

Cases	$C_{\text{part},x}$(nm)	$A_{\text{part},x}$(nm)	$C_{\text{part},y}$(nm)	$C_{\text{latt},y}$(nm)	$A_{\text{latt},y}$(nm)	$C_{\text{latt},y}$(nm)
Fig. 3a ($a = 150$ nm, $p = 300$ nm)	-280	155	0	-165	150	-115
Fig. 3b ($a = 150$ nm, $\eta = 1.5$)	-195	115	-10	-200	140	-115
Fig. 3c ($p = 300$ nm, $\eta = 1.5$)	-180	90	-40	-180	130	-140

Table S2. Comparison of the maximum values of the relative amendments to the resonant wavelengths for the three types of metasurface studied in this work and three directions: along x-axis ($\Delta\lambda_x^{\max}$), along y-axis ($\Delta\lambda_y^{\max}$) and along diagonal direction ($\Delta\lambda_d^{\max}$).

References

1. Yermakov, O. Y. *et al.* Effective surface conductivity of optical hyperbolic metasurfaces: from far-field characterization to surface wave analysis. *Sci. Rep.* **8**, 14135 (2018).
2. Hrinchenko, A. & Yermakov, O. Designing optical hyperbolic metasurfaces based on gold nanodisks. *J. Phys. D: Appl. Phys.* **56**, 465105 (2023).
